# Utilization of Health Care Services for Childhood Morbidity and Associated Factors in India: A National Cross-Sectional Household Survey

**DOI:** 10.1371/journal.pone.0051904

**Published:** 2012-12-19

**Authors:** Chandrashekhar T. Sreeramareddy, T. N. Sathyanarayana, H. N. Harsha Kumar

**Affiliations:** 1 Department of Clinical Sciences, Faculty of Medicine and Health Sciences, University Tunku Abdul Rahman, Sungai Long, Malaysia; 2 Indian Institute of Public Health, Bangalore Campus, Public Health Foundation of India, Bangalore, India; 3 Department of Community Medicine, Kasturba Medical College, Mangalore, India; Kenya Medical Research Institute - Wellcome Trust Research Programme, Kenya

## Abstract

**Background:**

Information about utilization of health services and associated factors are useful for improving service delivery to achieve universal health coverage.

**Methods:**

Data on a sample of ever-married women from India Demographic and Health survey 2005–06 was used. Mothers of children aged 0–59 months were asked about child’s illnesses and type of health facilities where treatment was given during 15 days prior to the survey date. Type of health facilities were grouped as informal provider, public provider and private provider. Factors associated with utilization of health services for diarrhea and fever/cough was assessed according to Andersen’s health behavior model. Multinomial logistic regression analyses were done considering sampling weights for complex sampling design.

**Results:**

A total of 48,679 of ever-married women reported that 9.1% 14.8% and 17.67% of their children had diarrhea, fever and cough respectively. Nearly one-third of the children with diarrhea and fever/cough did not receive any treatment. Two-thirds of children who received treatment were from private health care providers (HCPs). Among predisposing factors, children aged 1–2 years and those born at health facility (public/private) were more likely to be taken to any type of HCP during illness. Among enabling factors, as compared to poorer household, wealthier households were 2.5 times more likely to choose private HCPs for any illness. Children in rural areas were likely to be taken to any type of HCP for diarrhea but rural children were less likely to utilize private HCP for fever/cough. ‘Need’ factors i.e. children having severe symptoms were 2–3 times more likely to be taken to any type of HCP.

**Conclusion:**

Private HCPs were preferred for treatment of childhood illnesses. Involvement of private HCPs may be considered while planning child health programs. Health insurance scheme for childhood illnesses may to protect economically weaker sections from out-of-pocket health expenditure during child illness.

## Background

The Millennium Development Goal (MDG 4) aims to reduce child mortality (< five years old) by two-thirds between 1990 and 2015 [1], [2]. However, progress has been very slow in 60 countries where the child mortality rates are high [2], [3]. If currently available cost-effective interventions reach all the children living in low-and middle-income countries (LMICs), a majority of child deaths can be prevented [4], [5]. Acute Respiratory Infections (ARI) and Acute Diarrheal Diseases (ADD) are the most common acute childhood illness [6]–[8] and are major contributors to child mortality worldwide [9], [10]. The World Health Organization (WHO) estimates that seeking prompt and appropriate care during episodes of ARI and ADD could reduce child deaths by nearly 30% [11]. Therefore, Integrated Management of Childhood Illness (IMCI) emphasizes about improvement of families’ care seeking behavior in addition to improving providers’ skills in managing childhood illnesses [12]. One of the reasons for slow progress in achieving MDG-4 in many LMICs is the socio-economic inequities existing in these countries. These inequities may also affect access to and utilization of available health care services [13]–[15], time taken in seeking of medical care as well as selection of appropriate health care provider [16]–[21] for acute childhood illnesses. Studies from various countries suggest that health care seeking is inappropriate and health services are often under-utilized during childhood illnesses [13], [15], [20], [24], [26]. Studies have reported that high cost of treatment is a major deterrent to seek care [21], [27]–[29]. Mothers’ perceptions about symptoms and their severity [19], [30]–[34], mothers’ beliefs about childhood illnesses [27], [35] and mothers’ ability to recognize the danger signs [16], [17], [36] are some important factors determining health care-seeking behavior or utilization of health care services. Studies on utilization of health services during childhood illness have reported that a significant proportion of children are not taken for medical care [37], child’s gender plays a role in illness reporting [38], decision to choose health care provider [39] and private sector health services are preferred over public sector [21], [29], [40].

India is one among the 60 LMICs where reduction of child mortality rate has not progressed steadily towards achieving MDG-4. In India, infant mortality rate (IMR) and under-five mortality rate (U5MR) were 50 and 64 respectively per 1000 births during the year 2009 according to sample registration system (SRS) in India [22]. One of the reasons for slow decline in child mortality in India could be unequal distribution of healthcare resources and difficulties in access to health care [13]. Though a few studies have reported about utilization of health services for sick newborns from urban poor in Lucknow, northern India [23], childhood illness in rural Bihar [24], and Kerala state, southern India [25], there are no published country level reports about type of health services utilized (informal, public and private health care providers) for childhood illnesses. Our study would provide information about utilization of child health services at national level, which would be useful for policy making.

**Figure 1:**
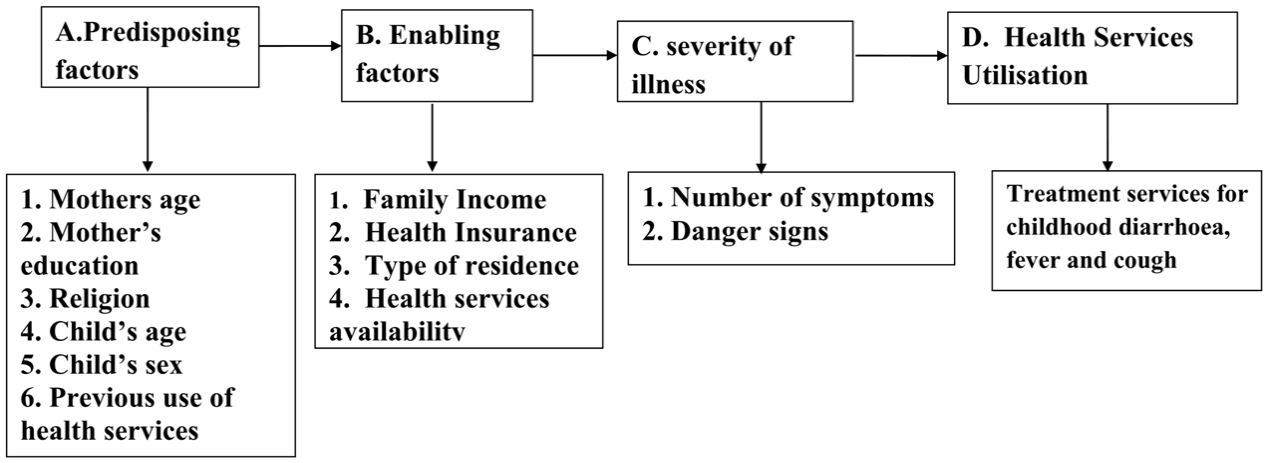
Conceptual framework for health care seeking behavior.

During the past two decades, social scientists and epidemiologists have emphasized that studies about health care seeking behavior and utilization of health services will provide good understanding about factors which may have programmatic and policy implications. Therefore such studies provide important information to the policy makers for designing strategies aimed to improve health care delivery [26]. Andersen’s health behavior model is widely accepted and used to study the determinants of health services utilization. We used this model as a conceptual framework for our analyses. Andersen’s model is based on three domains namely predisposing (demographic and social) factors, enabling (economic) factors, health system and ‘need’ factors as shown in figure 1. According to Andersen’s model, health services utilization is a sequential and conditional function of these three sets of factors. Predisposing factors will reflect the families which are likely to use health services while enabling factors are those which promote or hinder health service use. The first two sets of factors are not sufficient until the family perceives the severity of the illness. This is called as ‘need’ factor which is the most immediate reason for health service use according to Andersen [27]. The objectives of our analyses were 1) to report the type of health services from where medical treatment was provided during acute childhood illness and 2) to examine the factors associated with utilization of health services for childhood illness according to Andersen’s health behavior model (predisposing factors mother’s age, education, and religion, child’s age and sex, and previous use of health services; enabling factors family income, health insurance, urban or rural residence and availability of health services; need factors number of symptoms and presence of danger signs) [27].

## Methods

### Ethics Statement

The standard protocols, data collection tools and procedures for Demographic and Health Survey (DHS) were reviewed by Independent review boards (IRBs) of IPPS and ORC Macro International and approval was provided. During the survey, interviewers informed that the participation in the survey was voluntary. They were also assured about confidentiality of the information to be provided and could opt not to answer any of the questions during the interview. Informed consent was obtained from each survey participant.

### Study Setting

India has diversified health care system in which both public sector, private sector health care providers (HCPs) exist and HCPs practice both allopathic and indigenous systems (Ayurveda, Unani, Siddha) of medicine. Private sector health facilities provide mainly curative services, except for vaccinations, family planning services, and preventive checkups for chronic conditions while public sector health facilities provide both preventive and curative services. Public sector health facilities comprise of a wide network of primary health centers, sub centers, community health centers, district hospitals and tertiary care hospitals including teaching hospitals affiliated to medical schools. The preventive care services for women and children, and disease control programs are implemented through public sector health facilities. Though Government of India (GoI) is committed to provide universal and affordable health care services, the private health sector still dominates in terms of utilization rates with 80% of all outpatient visits and 60% of all hospital admissions being in private sector. Up to 70% of the health expenditure is out-of-pocket which throws 4% of the population into poverty [28], [29].

### Source of Data

In India, DHS is also known as National Family and Health Survey (NFHS). NFHS-III was conducted during the time period November 2005 to August 2006 under scientific and administrative supervision of the International Institute for Population Sciences (IPPS), Mumbai and ORC (Opinion Research Corp.) Macro International. The DHS team of trained interviewers collected the data about demography, socio-economic status, health examination, biomarkers and health related behavior from a nationally representative sample of households. In each of the selected households all eligible men and women were interviewed and health examination was done. Data were collected according to a standard protocol of DHS which had three core survey questionnaires for 1) Household details, 2) Women and 3) Men. These questionnaires used in all 29 states of India were translated into 18 local languages. After field testing the questionnaires were back-translated into English. In each state, the questionnaires used were bilingual i.e. questions were in the principal language of the state and English. Questionnaires were administered either in English or the principal language of the state or a preferred language of the household to minimize language barriers.

### Sampling Method

**Figure 2:**
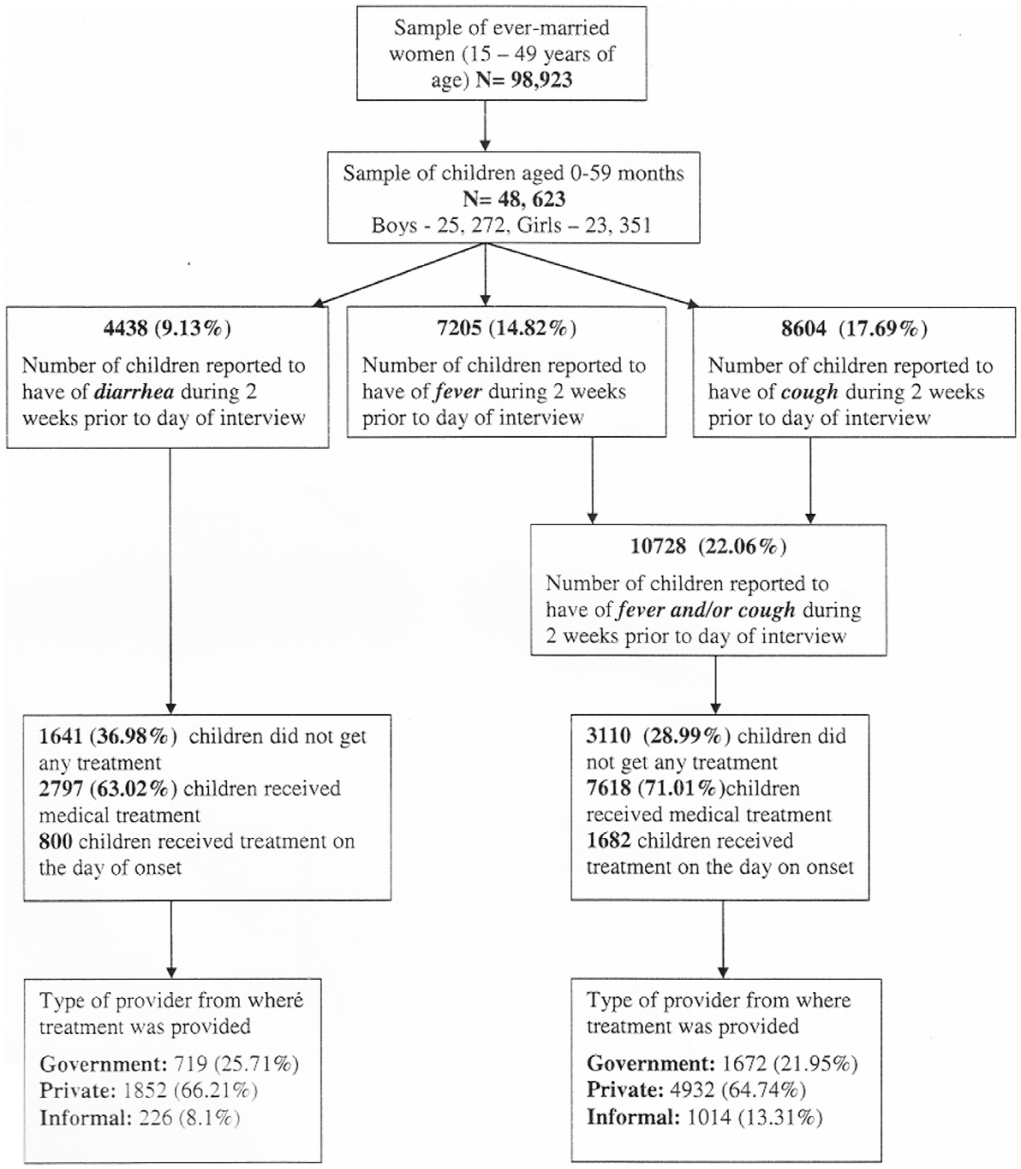
Flow chart for mothers’ reports child’s illness and health services utilization.

India DHS 2005/06 used a stratified, multistage cluster sampling method to obtain a nationally representative sample of households. The households were selected by two-stage probability proportional to size (PPS) method in rural areas and three stage PPS sampling method in urban areas and same sampling design was used in all29 states of India. Geographic sampling units were villages in rural areas and census blocks/wards in urban areas. A random sampling method with household as primary sampling unit (PSU) was undertaken in chosen geographic sampling units. Since PSU was a household, a national household weighing factor was used to maximize representativeness of the sample. Within each selected household, all ever married women aged between 15 and 49 years were eligible to be respondents for the survey. The sample size for India DHS 2005–06 was 109,041 households. The final sample obtained for our analyses are given in figure 2. More details about sampling design, training of the survey team, survey management and quality control measures are separately documented in the country reports published by ORC Macro International [30].

### Outcome Variables

Children aged 0–59 months (i.e. <5 years) who had suffered from episodes of diarrhea, fever and/or cough were included for our analyses. Outcome variables were obtained from the responses given by the respondents for following questions about three main symptoms of common acute childhood illnesses:

1. Has (name of the child) had diarrhea in the last 2 weeks?
2. Has (name of the child) been ill with a fever at any time in the last 2 weeks?
3. Has (name of the child) been ill with a cough at any time in the last 2 weeks?

If the response was ‘yes’ for any of the above three questions the following question was asked.

1. Did you seek advice or treatment for the illness from any source?

If the response was ‘yes’, three further questions were asked.

1. Where did you seek advice or treatment? Any other?
2. Where did you first seek advice or treatment?
3. How many days after the illness began did you first seek advice or treatment for (name of the child)?

The options given for “where did you seek advice or treatment?” were classified as public sector, private sector, non-governmental organization (NGO), trust hospital, and others (shop, friend). We grouped NGO/trust hospital into private sector, and treatment from pharmacy, practitioners of indigenous medicine and traditional healers as ‘informal sector’ though original DHS questionnaire had grouped them as separate categories. For our analyses, we combined ‘no treatment given’ with ‘informal sector’. Thus we had following three categories: 1) no treatment/informal sector, 2) public sector, and 3) private sector. Though fever and cough were reported as separate symptoms, respondents were asked as “where did you seek advice or treatment?” for both symptoms together to indicate these symptoms as Acute Respiratory Infections (ARIs). We used data about episodes of diarrhea and fever/cough as two different acute childhood illnesses to perform separate analysis about utilization of health care services for diarrhea and fever/cough.

### Explanatory Variables

Predisposing factors included for our analyses were age, religion and educational attainment of the mother, age and sex of the child, and previous use of health services (i.e. place of childbirth which was classified as home, private sector and public sector). We used wealth quintiles and possession of BPL (below poverty line) card by the family or possession of health scheme or insurance by any family member as enabling factors. Socio-demographic information was obtained for each eligible woman during the interview in the woman’s questionnaire. Age and sex of child and place of delivery was obtained from the birth history. Wealth index, a relative index of household wealth was calculated based on a standard set of household assets, dwelling characteristics and ownership of consumer items according to the interviewer’s observation. The individuals were then ranked on the basis of household score calculated using the above items. Later individuals were divided into quintiles, where the first quintile is the poorest 20% of the households and fifth quintile is the wealthiest 20% of the households. In woman’s questionnaire respondent was asked if the household was given BPL card and if any person in the house possessed any type of health insurance or a scheme. For our analysis, we grouped mother’s age in years (15–24, 25–34 and 35–49), child’s age in years (<1, 1–2 and 3–5), religion (Hindu, Muslim, Christian and other) mother’s education (no education, primary level, secondary and higher) place of delivery (home, private hospital and public hospital). For health system factors, we included type of residence i.e. urban or rural as a proxy measure for availability of health services. Respondents were asked about various factors that could prevent women from getting medical advice or treatment for themselves. The responses were rated as ‘a big problem’, ‘a small problem’, and ‘no problem’. We included the responses to the following three items as measures of accessibility: 1) ‘distance to the health facility’, 2) ‘concern that there may not be any health provider’, and 3) ‘concern that there may be no drugs available’. In addition to the episodes of diarrhea, fever and cough, the respondents were also asked about symptoms which could indicate serious illness. Symptoms of severe illness i.e. presence of blood in the stools, rapid breathing, problem in the chest, and blocked/running nose were used as ‘need’ factors.

### Data Analysis

Statistical analyses were carried out in SPSS (Statistical Package for Social Sciences) version-17. Prevalence rates for episodes of diarrhea, fever and cough were calculated. Proportion of children with episodes of diarrhea and fever/cough who received medical treatment was calculated according to type of provider. Factors associated with utilization of health services (no care/informal care, public HCP and private HCP) for diarrhea and fever/cough were assessed. Multinomial logistic regression analyses were done using 'complex samples' option in SPSS to account for multistage sampling used in DHS. To assess the fixed effects of explanatory variables on health service utilization, multinomial logistic regression modeling was done in which conceptually linked explanatory variables were entered into the model as blocks. In the first step, predisposing factors were entered into the model, followed by enabling factors and lastly the health system and need factors. For the outcome variable i.e. utilization of health services, no care/informal care was used as reference category against utilization of private sector and public sector HCPs. Adjusted odds ratios (aOR) and their 95% confidence intervals (95% CIs) were reported for all the models. A p-value less than 0.05 were considered as significant.

## Results

The survey sampled a total of 116,652 households of which 109,070 completed the survey giving a household response rate of 93.5%. The number of eligible women in these households was 131,596 of whom 94.5% completed the survey. These 98,923 ever married women aged between 15 and 49 years had 48, 623 children who were aged between 0 and 59 months. The prevalence of diarrhea, fever and cough reported by mothers during two weeks prior to the day of interview was 9.1%, 14.8% and 17.7% respectively. The proportion of children, who did not receive any type of medical treatment during an episode of diarrhea and fever/cough, was 36.9% and 28.9% respectively. Among those who received any medical treatment for diarrhea, the proportion of children who received treatment from private HCPs was 66.2% and from public HCPs was 25.7%. The proportion of children who received treatment for fever/cough from private HCPs was 64.7% and public HCPs was 21.9% (figure 2).

The background demographic characteristics of the sample included for our analyses and utilization of health services for episodes of diarrhea and fever/cough are shown in tables 1 and 2 respectively. Utilization of health services during episodes of diarrhea and fever/cough was compared with selected demographic and other explanatory variables. The differences in proportion of mothers utilizing public and private HCPs according to explanatory variables were significant for all except for mother’s age, possession of health scheme/insurance, and sex of the child (table 1). For fever/cough, the proportion of mothers utilizing public HCPs and private HCPs showed significant differences for all explanatory variables (table 2).

**Table 1:** Type of provider where treatment was sought for diarrhea by demographic & economic factors.

	Number ofparticipants	No treatment orinformal care	Public provider	Private provider
**Mother’s age** †				
15–24	1867	830 (49.5)	283 (15.2)	754 (40.4)
25–34	2210	1007 (45.6)	317 (14.3)	886 (40.1)
35–49	321	169 (52.6)	40 (12.5)	112 (34.9)
**Mother’s education** *				
No education	1724	845 (49.0)	226 (13.1)	653 (37.9)
Primary	657	343 (52.2)	100 (15.2)	214 (32.6)
Secondary	1767	734 (41.5)	292 (16.5)	741 (41.9)
Higher	224	84 (37.5)	22 (9.8)	118 (52.7)
**Mother’s religion** *				
Hindu	2987	1290 (43.2)	414 (13.9)	1283 (42.9)
Muslim	805	343 (42.6)	116 (14.4)	346 (43.0)
Christian & others	642	371 (57.8)	110 (17.1)	161 (25.1)
**Type of residence** *				
Urban	1689	686 (40.6)	210 (12.4)	793 (47.0)
Rural	2749	1320 (48.0)	430 (15.6)	999 (36.4)
**Wealth quintiles** *				
Poorest	767	429 (55.9)	98 (12.8)	240 (31.3)
Poorer	812	418 (51.5)	127 (15.6)	267 (32.9)
Middle	968	460 (47.5)	174 (18.0)	334 (34.5)
Richer	1028	423 (41.1)	150 (14.6)	455 (44.3)
Richest	863	276 (32.0)	91 (10.5)	496 (57.5)
**Age of the child** *				
<1 year	1377	613 (44.5)	177 (12.9)	587 (42.6)
1–2 years	1302	520 (39.9)	207 (15.9)	575 (44.2)
3–5 years	1759	873 (49.6)	256 (14.6)	630 (35.8)
**Sex of the child** †				
Male	2425	1065 (43.9)	359 (14.8)	1001 (41.3)
Female	2013	941 (46.7)	281 (13.0)	791 (39.3)
**Place of birth** *				
Home	2389	1228 (51.4)	313 (13.1)	848 (35.5)
Public hospital	1156	490 (42.4)	273 (23.6)	393 (34.0)
Private hospital	893	288 (32.3)	54 (6.0)	551 (61.7)
**Health scheme/insurance** †				
None	3380	1533 (45.4)	466 (13.8)	1381 (40.8)
Yes	1054	471 (44.7)	174 (16.5)	409 (38.9)

* For these comparisons p-value was less than 0.001.

† For these comparison p-values was ≥0.05.

**Table 2:** Type of provider where treatment was sought for fever/cough by demographic & economic factors.

	Number ofparticipants	No/informalcare	Public provider	Private provider
**Mother’s age** *				
15–24	4324	1743 (40.3)	602 (13.9)	1979 (45.5)
25–34	5524	2279 (41.3)	776 (14.1)	2469 (44.6)
35–49	880	423 (48.1)	93 (10.6)	364 (41.3)
**Mother’s education** *				
No education	3968	1854 (46.7)	478 (12.1)	1636 (41.2)
Primary	1676	799 (47.7)	239 (14.3)	638 (38.0)
Secondary	4304	1568 (36.4)	678 (15.8)	2058 (47.8)
Higher	780	224 (28.7)	76 (9.7)	480 (61.6)
**Religion** *				
Hindu	7196	2914 (40.5)	922 (12.8)	3360 (46.7)
Muslim	2101	763 (36.3)	296 (14.1)	1042 (49.6)
Christian & others	1420	760 (53.5)	252 (17.6)	408 (28.7)
**Type of residence** *				
Urban	4125	1422 (34.5)	499 (12.1)	2204 (45.8)
Rural	6603	3023 (45.8)	972 (14.7)	2608 (39.5)
**Wealth quintiles** *				
Poorest	1768	957 (54.1)	183 (10.4)	628 (35.5)
Poorer	2013	1018 (50.6)	284 (14.1)	711 (35.3)
Middle	2292	977 (42.6)	412 (18.0)	903 (39.4)
Richer	2435	883 (36.3)	346 (14.2)	1206 (49.5)
Richest	2220	610 (27.5)	246 (11.1)	1364 (28.3)
**Age of the child** *				
<1 year	2390	935 (39.1)	322 (13.5)	1133 (47.4)
1–2 years	2516	985 (39.1)	347 (13.8)	1184 (47.1)
3–5 years	5822	2525 (43.4)	802 (13.8)	2495 (42.9)
**Sex of the child** *				
Male	5712	2257 (39.5)	795 (13.9)	2660 (46.6)
Female	5016	2188 (43.6)	676 (13.5)	2152 (42.9)
**Place of delivery** *				
Home	5660	2731 (48.2)	671 (11.9)	2258 (39.9)
Public hospital	2741	1017 (37.1)	644 (23.5)	1080 (39.4)
Private hospital	2327	697 (30.0)	156 (6.7)	1474 (63.3)
**Health scheme/insurance** *				
None	8142	3346 (41.1)	1049 (12.9)	3747 (46.0)
Yes	2581	1095 (42.4)	422 (16.4)	1064 (41.2)

* For these comparisons p-value was less than 0.001.

Among predisposing factors, child’s age, gender and place of delivery were associated with utilization of health services. Children aged 1–2 years had higher odds of being taken to any type of HCP (aOR 1.54, 95% CIs 1.12, 2.13) for treatment of diarrhea as compared to children aged <1 year. As compared to the children born at home, children who were born at private facility had higher odds of being taken to private HCPs for treatment of either diarrhea or fever/cough (aOR 1.50, 95% CIs 1.18 1.92 and aOR 1.49, 95% CIs 1.24 1.81). There was similar association for those children who were born in public sector health facilities. Female children had lower odds of being taken to public HCP for treatment of diarrhea (tables 3 and 4).

**Table 3:** Factors associated with choice of type of health care provider for treatment of diarrhea.

	Model 1			Model 2		Model 3	
	No care vs.Public		No care vs.Private	No care vs.Public	No care vs.Private	No care vs.Public	No care vs.Private
	OR (95% CIs)		OR (95% CIs)	OR (95% CIs)	OR (95% CIs)	OR (95% CIs)	OR (95% CIs)
**Age of the mother**							
15–24	1		1	1	1	1	1
25–34	1.02 (0.78,1.32)		0.88 (0.73, 1.06)	1.02 (0.79, 1.32)	0.69 (0.40, 1.18)	1.04 (0.80, 1.36)	0.87 (0.72, 1.05)
35–49	0.69 (0.40,1.18)		0.93 (0.65, 1.38)	0.86 (0.71, 1.04)	0.91 (0.63, 1.30)	0.65 (0.38, 1.12)	0.92 (0.64, 1.32)
**Mother’s education**							
No education	1		1	1	1	1	1
Primary	1.17 (0.81, 1.69)		1.02 (0.78, 1.33)	1.21 (0.83, 1.76)	0.93 (0.71, 1.22)	1.17 (0.80, 1.17)	0.92 (0.69, 1.20)
Secondary	1.13 (0.82, 1.55)		1.23 (0.99, 1.52)	1.17 (0.83, 1.67)	0.93 (0.74, 1.85)	1.13 (0.79, 1.60)	0.91 (0.72, 1.15)
Higher	0.57 (0.23, 1.15)		**2.08 (1.29, 3.35)**	0.56 (0.24, 1.31)	1.26 (0.75, 2.13)	0.49 (0.21, 1.17)	1.16 (0.68, 1.99)
**Religion**							
Hindu	1		1	1	1	1	1
Muslim	1.08 (0.76, 1.54)		1.19 (0.92, 1.55)	1.08 (0.76, 1.54)	1.14 (0.88, 1.47)	1.08 (0.75, 1.55)	1.10 (0.86, 1.42)
Christian & others	1.22 (0.68, 2.17)		0.98 (0.69, 1.41)	1.21 (0.68, 2.16)	0.95 (0.67, 1.34)	1.23 (0.69, 2.21)	0.96 (0.67, 1.36)
**Place of delivery**							
Home	1		1	1	1	1	1
Public hospital	**2.22 (1.65, 2.98)**		0.89 (0.71, 1.11)	**2.26 (1.66, 3.11)**	**0.76 (0.60,0.96)**	**2.35 (1.72, 3.21)**	**0.78 (0.61, 0.98)**
Private hospital	0.85 (0.55, 1.32)		**1.86 (1.48, 2.34)**	0.88 (0.56, 1.39)	**1.51 (1.18, 1.90)**	0.86 (0.54, 1.37)	**1.50 (1.18, 1.92)**
**Age of the child**							
<1 year	1		1	1	1	1	1
1–2 Year	**1.64 (1.19, 2.26)**		**1.37 (1.09, 1.70)**	**1.63 (1.18, 2.25)**	**1.38 (1.10, 1.72)**	**1.54 (1.12, 2.13)**	**1.34 (1.07, 1.67)**
>3 years	1.20 (0.88, 1.64)		0.87 (0.72, 1.07)	1.19 (0.87, 1.63)	0.86 (0.71, 1.06)	1.14 (0.83, 1.56)	0.83 (0.67, 1.02)
**Sex of the child**							
Male	1		1	1	1	1	1
Female	**0.78 (0.62, 0.99)**		0.90 (0.77, 1.06)	**0.78 (0.62, 0.99)**	0.91 (0.77, 1.07)	**0.77 (0.61, 0.98)**	0.91 (0.77, 1.07)
**Wealth Quintiles**							
Poorest	1		1	1	1	1	1
Poorer				0.91 (0.61, 1.35)	**1.33 (1.02, 1.75)**	0.93 (0.63, 1.39)	**1.32 (1.00, 1.73)**
Middle				0.97 (0.66, 1.44)	**1.54 (1.16, 2.06)**	1.03 (0.69, 1.55)	**1.54 (1.15, 2.08)**
Richer				0.90 (0.58, 1.41)	**1.98 (1.46, 2.68)**	0.99 (0.62, 1.61)	**1.99 (1.44, 2.78)**
Richest				0.92 (0.54, 1.57)	**2.82 (1.94, 4.12)**	1.03 (0.56, 1.87)	**2.80 (1.85, 4.25)**
**Health Scheme/Insurance**							
None				1	1	1	1
Yes				1.04 (0.77, 1.39)	1.18 (0.96, 1.47)	1.03 (0.76, 1.34)	1.18 (0.95, 1.47)
**Distance of facility**							
No problem						1	1
Not a big problem						0.79 (0.56, 1.14)	0.81 (0.64, 1.03)
Big Problem						**0.66 (0.47, 0.93)**	0.85 (0.66, 1.10)
**No provider**							
No problem						1	1
Not a big problem						1.05 (0.66, 1.68)	0.82 (0.59, 1.15)
Big Problem						0.78 (0.49, 1.23)	1.02 (0.74, 1.40)
**No drugs**							
No problem						1	1
Not a big problem						1.27 (0.79, 2.06)	0.94 (0.68, 1.30)
Big Problem						**1.57 (1.01, 2.43)**	0.84 (0.62, 1.14)
**Type of residence**							
Urban						1	1
Rural						1.15 (0.81, 1.65)	1.23 (0.97, 1.55)
**Blood in stools**							
No						1	1
Yes						1.40 (0.89, 2.19)	**1.55 (1.14, 2.01)**

**Table 4:** Factors associated with choice of type of health care provider for treatment of fever and/or cough.

	Model 1		Model 2		Model 3	
	No care vs. Public	No care vs. Private	No care vs. Public	No care vs. Private	No care vs. Public	No care vs. Private
	OR (95% CIs)	OR (95% CIs)	OR (95% CIs)	OR (95% CIs)	OR (95% CIs)	OR (95% CIs)
**Age of the mother**						
15–24	1	1	1	1	1	1
25–34	0.96 (0.81, 1.19)	0.95 (0.84, 1.08)	0.98 (0.81, 1.20)	0.92 (0.81, 1.04)	0.98 (0.79, 1.22)	0.92 (0.79, 1.06)
35–49	0.91 (0.63, 1.31)	0.99 (0.78, 1.27)	0.88 (0.61, 1.29)	0.97 (0.76, 1.24)	0.85 (0.56, 1.29)	0.96 (0.73, 1.27)
**Mother’s education**						
No education	1	1	1	1	1	1
Primary	1.08 (0.83, 1.42)	0.99 (0.84, 1.18)	1.02 (0.77, 1.35)	0.91 (0.76, 1.08)	0.93 (0.68, 1.27)	0.87 (0.72, 1.05)
Secondary	**1.26 (1.00, 1.58)**	**1.32 (1.13, 1.54)**	1.12 (0.87, 1.44)	0.97 (0.83, 1.51)	1.09 (0.82, 1.44)	0.99 (0.82, 1.19)
Higher	1.14 (0.73, 1.79)	**1.64 (1.23, 2.19)**	0.99 (0.61, 1.63)	0.99 (0.72, 1.37)	1.09 (0.63, 1.89)	0.99 (0.67, 1.45)
**Religion**						
Hindu	1	1	1	1	1	1
Muslim	1.16 (0.92, 1.47)	1.26 (1.07, 1.48)	1.16 (0.91, 1.48)	1.21 (1.04, 1.43)	1.17 (0.89, 1.54)	1.09 (0.91, 1.31)
Christian & others	1.08 (0.77, 1.51)	1.01 (0.77, 1.32)	1.07 (0.76, 1.50)	0.94 (0.73, 1.22)	1.05 (0.70, 1.59)	1.04 (0.78, 1.40)
**Place of delivery**						
Home	1	1	1	1	1	1
Public hospital	**2.36 (1.89, 2.95)**	1.00 (0.86, 1.17)	**2.22 (1.78, 2.77)**	0.88 (0.76, 1.03)	**2.27 (1.77, 2.91)**	0.90 (0.75, 1.07)
Private hospital	0.84 (0.64, 1.12)	**1.79 (1.54, 2.10)**	0.77 (0.58, 1.04)	**1.41 (1.19, 1.67)**	0.82 (0.59, 1.13)	**1.49 (1.24, 1.81)**
**Age of the child**						
<1 year	1	1	1	1	1	1
1–3 Year	1.01 (0.78, 1.29)	0.97 (0.83, 1.15)	1.00 (0.78, 1.28)	0.96 (0.82, 1.14)	1.07 (0.82, 1.40)	0.91 (0.75, 1.11)
>3 years	1.08 (0.88, 1.34)	**0.84 (0.70, 0.92)**	1.05 (0.85, 1.29)	**0.78 (0.68, 0.89)**	1.20 (0.87, 1.38)	**0.78 (0.67, 0.93)**
**Sex of the child**						
Male	1	1	1	1	1	1
Female	0.94 (0.81, 1.10)	**0.87 (0.78, 0.97)**	0.94 (0.81, 1.10)	**0.88 (0.79, 0.98)**	0.87 (0.74, 1.04)	0.89 (0.79, 1.02)
**Wealth Quintiles**						
Poorest	1	1	1	1	1	1
Poorer			1.22 (0.92, 1.61)	**1.19 (1.02, 1.42)**	1.26 (0.91, 1.74)	**1.27 (1.05, 1.53)**
Middle			**1.63 (1.22, 2.18)**	**1.51 (1.26, 1.79)**	**1.68 (1.21, 2.34)**	**1.49 (1.21, 1.85)**
Richer			**1.42 (1.02, 1.97)**	**1.98 (1.61, 2.44)**	1.45 (0.99, 2.11)	**1.82 (1.42, 2.34)**
Richest			**1.59 (1.08, 2.33)**	**2.71 (2.13, 3.44)**	**1.84 (1.15, 2.94)**	**2.42 (1.78, 2.30)**
**Health Scheme/Insurance**						
None			1	1	1	1
Yes			**1.36 (1.11, 1.66)**	0.98 (0.85, 1.13)	**1.40 (1.11, 1.75)**	1.02 (0.87, 1.19)
**Distance of facility**						
No problem					1	1
Not a big problem					1.02 (0.77, 1.35)	0.92 (0.77, 1.20)
Big Problem					0.86 (0.66, 1.13)	0.97 (0.82, 1.16)
**No provider**						
No problem					1	1
Not a big problem					1.28 (0.86, 1.89)	0.91 (0.70, 1.19)
Big Problem					1.12 (0.79, 1.59)	1.11 (0.89, 1.39)
**No drugs**						
No problem					1	1
Not a big problem					0.73 (0.48, 1.11)	**0.72 (0.54, 0.95)**
Big Problem					1.12 (0.81, 1.57)	**0.76 (0.61, 0.94)**
**Type of residence**						
Urban					1	1
Rural					0.97 (0.74, 1.28)	**0.81 (0.68, 0.97)**
**Other symptoms**						
None					1	1
Blocked nose/chest					**1.89 (1.49, 2.42)**	**2.17 (1.84, 2.55)**
Difficult breathing					**2.65 (2.04, 3.46)**	**4.11 (3.43, 4.93)**

Among enabling factors, household wealth was positively associated with use of private HCPs for treatment of both diarrhea and fever/cough. For treatment of diarrhea, women belonging to richest households had higher odds of taking their children to a private HCPs (aOR 2.80, 95% CIs 1.85, 4.25) as compared to women from poorest households and the association was similar for treatment of fever/cough (aOR 2.42, 95% CIs 1.78 2.30). Possession of health scheme/insurance was positively associated with use of public HCPs for fever/cough (aOR 1.40, 95% CIs 1.11, 1.75) (tables 3 and 4).

Urban/rural type of residence, mother’s perception of distance of health facility, non-availability of drugs as a ‘problem’ were associated with use of public HCP for treatment of diarrhea whereas these factors were associated with use of private HCPs for treatment of fever/cough. Presence of blood in the stools was positively associated with the use of private HCPs (aOR 1.55, 95% CIs 1.14, 2.01) whereas symptoms of ‘blocked nose/chest’ and ‘difficult breathing’ was positively associated with use of either public HCP (aOR 2.65, 95% CIs 2.04, 3.46) or private HCP (aOR 4.11, 95% CIs 3.43, 4.93) (tables 3 and 4).

## Discussion

Our report brings into light some important issues about delivery of curative services for two important causes of childhood mortality. The most important finding from our analyses was that more than a third of children who were reported to be having diarrhea and fever/cough did not receive any medical treatment. Two-thirds of the children received treatment for their illnesses from private HCPs. Children who were younger and having severe symptoms were likely to be taken for medical treatment. Place of delivery was an important factor associated with the type of HCP chosen. Children from wealthier households were likely to be treated by private HCPs for both diarrhea and fever/cough.

All the interpretations of our results should be made considering the following strengths and limitations. The main strength of our analyses was generalisability of our results to the entire nation although we did not analyze for any regional or state level variations. A standardized methodology used by DHS enabled us to make comparisons with similar data from other countries. The strength was that we used a conceptual framework based on Andersen’s health behavior model which is a widely accepted method for assessment of factors associated with health services utilization. Some of the potential limitations include selection bias, recall bias and non-availability of some variables in DHS data. The final sample used in our analyses included only those care givers who reported their children to be ill during previous two weeks. It is possible that those reported about illness may have been statistically different from those who did not report any illness. This may have lead to selection bias. For instance, children from poorest households have a higher risk of suffering from diarrhea or fever/cough than children from richer or richest household and are also less likely to receive any treatment. Mother’s self-report about child’s illness may not have been accurate due to recall bias and misconceptions about childhood illnesses. This may have lead to under-reporting of child illness. However, DHS did not have any means to objectively verify the reports of illness given by mothers. Mothers who did not report child’s illness may have been least educated and from poorest households. Nevertheless, we presume that the proportion of mothers who did not report about child’s illness may be very small. Family income which is an important determinant for health service utilization was not available in DHS data. Nevertheless, we used wealth index as a proxy measure for income. Wealth index has been shown to be relevant in developing countries [31]. Data about availability, and accessibility of health services which influence their utilization were not collected in DHS. However, we used type of residence and mother’s concern about health services as proxy for these explanatory variables. This may be the reason for lack of association of these factors with health service utilization. Furthermore, information about perceived quality of care and health beliefs which may affect utilization of health services was not available in DHS data.

A surprising finding was that nearly a third of children did not receive any medical treatment is supported by other reports based on DHS [32], [33]. However, for diarrhea, most mothers may have given Oral Rehydration Solutions (ORS) for which ORS sachets or home-made rehydration fluids, both of which are easily available. For fever and cough mothers may have given a trial with medicines available at home or herbal medicines or home remedies before visiting a HCP. Relatively lower rates of health services utilization may be a result of self-treatment which has been well documented in literature [19], [34]–[36]. Our analysis was only about type of HCP who treated the child but did not consider the treatment given at home. Therefore, it is possible that these figures may be an overestimate for both diarrhea and fever/cough. Moreover, our analyses showed that children are likely to be treated outside, if they had severe symptoms (blood in stools, difficult or rapid breathing, and blocked chest/nose). Studies from LMICs have reported that children are likely to be taken for medical treatment if mother perceived the illness as serious [19], [21], [25], [35], [37]–[40].

Among the predisposing factors, children aged 1–2 years had higher odds for receiving medical treatment as observed in a report from sub-Saharan Africa [19], [32]. Since previous experience of using health services may influence utilization of HCPs for childhood illness, place of birth was included in our analyses. The mothers who gave birth at a public facility had higher odds for choosing public HCP and mothers who gave birth in private hospitals had higher odds for choosing private HCP for treatment of their child’s illness. This observation goes in hand with the enabling factors i.e. ability to pay for services at private hospitals or mother preferred a health facility where she had given birth. For diarrhea, female children were less likely to get treatment from public HCP but the effect of gender bias was marginal. Gender bias against female child has been reported from some LMICs [21], [24], [36], [37], [40], [41]. However, there was no such bias for treatment of fever/cough in our study. Some studies have reported that gender bias does not exist for seeking health care for child illnesses [19], [25], [39]. Mothers’ education was not associated with treatment behavior for child illness though available literature suggests otherwise [19], [21], [26], [32].

Among enabling factors tested in the models, household wealth was positively associated with use of private HCPs for both diarrhea and fever/cough which is in conformity with many studies reported from LMICs [19]–[21], [24]–[26], [32], [37], [39]. However, for treatment of fever/cough, wealth index was not associated with use of public HCPs except for two wealth index categories which could not be explained from our data. Use of private HCPs showed a clear gradient across wealth quintiles with richer and richest having higher and highest odds for seeking treatment from private HCPs. Possession of health scheme/insurance by a family member was positively associated with use of public HCPs because most of them were holders of BPL (below poverty line) card. Though BPL card provides privileges for hospitalization in both publicly-funded and some private health facilities, choice of public HCP may be a direct result of economic status i.e. being below the poverty line [42]. Our argument is supported by results based on 61st round of Consumer Expenditure Survey (CES) (2004–05) done by National Sample Survey Organization (NSSO) of India which has reported that nearly half of BPL households had also made out-of-pocket payment for health care [43].

The need factor i.e. presence of severe symptoms was positively associated with use of any type of HCP highlights that ‘need’ is an important and immediate factor for use of HCP for treatment of childhood illnesses. This is in accordance with previous literature which have reported that the number of symptoms and care giver’s perceived severity of child’s illness are positively associated with prompt treatment [19], [21], [24]–[26], [37], [44]–[46]. Therefore, education of mothers/caregivers about danger signs has been included in IMCI strategy to improve caregiver’s health care seeking behavior. For fever/cough, private HCPs had lower odds of being consulted in rural areas but the same was not true for diarrhea. This could be due to lesser availability of private HCPs in rural areas or inability to pay fees to private HCPs by the rural poor [47]. Moreover, ORS sachets are widely available and mothers may have been well aware of oral rehydration therapy. Mothers who perceived that distance to health facility as a ‘big problem’ had lower odds for seeking treatment for diarrhea from a public HCP. Widespread availability of private clinics/hospitals in urban areas and some of the rural areas may be a reason for this. Our result about distance to health facility was not associated with utilization of health services for fever/cough. However one study has calculated travel time using geographic information systems and demonstrated that physical distance is an important determinant of utilization of health services [48]. The association of mother’s perception of ‘no provider’ and ‘no drugs’ with utilization of HCPs was contradictory for diarrhea and fever/cough. Our analyses was about type of HCPs where treatment was given but during the interview, the questions about these factors were not specifically pointed towards private or publicly funded health services.

### Policy Implications

Our analysis reveals the existing gaps in service delivery of child health interventions aimed at reducing child mortality from diarrhea diseases and respiratory infections. Nearly three-fourths of the population in India seek initial treatment for illness from private health sector testifies that gaps exist for delivery of child health interventions as well [13], [28], [42]. People are forced to make out-of-pocket expenditure by choosing large unregulated private health sector since public sector health facilities are largely inaccessible. Care-seeking behavior was far from adequate since only a quarter of sick children received medical treatment on the day illness started. Therefore, improvement of family and community practices as one of the component in IMNCI strategy is needed. However, the plan of up-scaling IMNCI should be considered cautiously, since two-thirds of the sick children had received treatment from a large unregulated private sector. IMNCI training is only provided to the health care providers in the publicly-funded health services. The GoI intends to involve private HCPs in health care delivery in its national population policy [49]. This policy may increase the provider supply but may not reach the vulnerable population groups particularly the economically weaker sections. Therefore, it has been suggested that health system reforms may be necessary for effective implementation of IMNCI or NRHM (National Rural Health Mission) [50]. Moreover, interventions aimed to improve utilization of health services should consider socio-economic development of the community in which they are to be implemented [32].

### Conclusions

Utilization of private HCP was consistently associated with wealth index and presence of severe symptoms was associated with utilization of both types of HCPs. Previous use of private health facility for delivery was associated with choosing private HCPs. Since private sector HCPs play an important role in treatment of acute childhood illness, health system reforms like regulation of private health sector and involvement of private HCPs in child health programs should be considered while program planning. National health insurance scheme must cover child illnesses to protect economically weaker sections from out-of-pocket health expenditure during visits to private HCPs.
